# Gastrointestinal stromal tumors: Fibrinogen levels are associated with prognosis of patients as blood-based biomarker

**DOI:** 10.1097/MD.0000000000010568

**Published:** 2018-04-27

**Authors:** Jing Lu, Shuangjiang Chen, Xuqi Li, Guanglin Qiu, Shicai He, Haijiang Wang, Libo Zhou, Yaheng Jing, Xiangming Che, Lin Fan

**Affiliations:** a Department of General Surgery, The First Affiliated Hospital, School of Medicine, Xi’an Jiaotong University, Xi’an Shaanxi; b Department of General Surgery, Ankang People's Hospital, Ankang Shaanxi, P.R. China.

**Keywords:** biomarker, coagulation factors, fibrinogen, gastrointestinal stromal tumors, prognosis

## Abstract

Improved prediction of prognosis for primary gastrointestinal stromal tumors (GISTs) after curative resection is an important goal in clinical practice. Coagulation factor of fibrinogen may inform prognosis of tumor patients as blood-based biomarker. Here, we aimed to analyze the prognostic value of fibrinogen levels in patients with GIST and to explore potential threshold of fibrinogen on postoperative clinical outcome.

A retrospective study was performed including data from 91 patients with newly diagnosed GISTs who underwent curative resection. Patients were followed-up for a median period of 2 years. Cox regression and competing risk analysis were applied to study the association between fibrinogen and risk of death or recurrence. Smoothing plot and threshold effect analysis were applied to learn the relationship further and explore potential threshold.

High levels of fibrinogen are associated with decreased overall survival (OS) and recurrence free survival (RFS) in patients with GISTs. We discovered a nonlinear relationship between levels of fibrinogen and the risk of death or recurrence. Further, we detected a threshold for fibrinogen (3.7 g/L) on the prognosis of GISTs patients. When fibrinogen was above the inflection point, the increase in fibrinogen levels was strongly associated with increase in the risk of death or recurrence.

Elevated fibrinogen can serve as an independent prognostic biomarker for a worse clinical outcome in GIST patients.

## 1 Introduction

Gastrointestinal stromal tumors (GISTs) are neoplasms originating mainly from the mesenchymal tissue of the gastrointestinal tract and seldom from other soft tissues in the abdominal cavity.^[1]^ GISTs may occur at any location along the gastrointestinal tract, though the most common sites being the stomach (50–60%) and the small intestine (30–35%).^[2]^ Surgical resection remains the standard treatment in localized stadium and should be carried out whenever feasible. GISTs’ malignant potential covers a wide spectrum ranging from small benign lesions to aggressive sarcomas, making GIST a particular type in all the tumors. These characteristics are associated with progression and tumor recurrence even after complete excision.^[3]^

Approximately, 75% to 80% of GISTs harbor kinase-activating gene mutations in KIT. Therefore the advent of imatinib, a selective inhibitor of the KIT receptor, has changed the GIST treatment paradigm as a targeted systemic treatment.^[4,5]^ In the present therapeutic setting, patients at intermediate or high risk of recurrence, should be considered for adjuvant imatinib treatment after resection.^[6,7]^ Accordingly, it's increasingly important to predict the prognosis of GISTs in order to administrate adjuvant therapy to the appropriate patients in time after surgery. Currently, the evaluation tool of the prognosis for GISTs is mainly based on the tumor-specific parameters such as tumor location, tumor size, mitotic rate, and also tumor rupture, among which Miettinen score being the most frequently used. Improving the prediction of recurrence free survival (RFS) in GIST patients after surgery is an important goal of research, because this can spare patients with low risk of recurrence from unnecessary treatment and direct adjuvant therapy to the ones with the highest clinical benefit.

Fibrinogen (FIB) is a coagulation factor which can be tested in plasma. And it has been reported that FIB is prognostic indicator for several malignancies, including cervical cancer,^[8]^ esophagus carcinoma,^[9]^ gastric cancer,^[10]^ and colorectal cancer.^[11]^ It is related to tumor stage, tumor prognosis, and lymph node involvement and contribute to cancer growth, progression, and metastasis.^[12]^ FIB may reflect multifactorial interactions between tumor growth and the hemostatic-fibrinolytic system in malignancy. However in GISTs, the potential of this easily-available biomarker as predictor of prognosis has not been explored. Our study aimed to analyze the prognostic value of FIB levels in patients with GIST and to explore potential threshold of FIB in relation to postoperative survival.

## 2 Materials and methods

### 2.1 Patient selection

**Figure 1:**
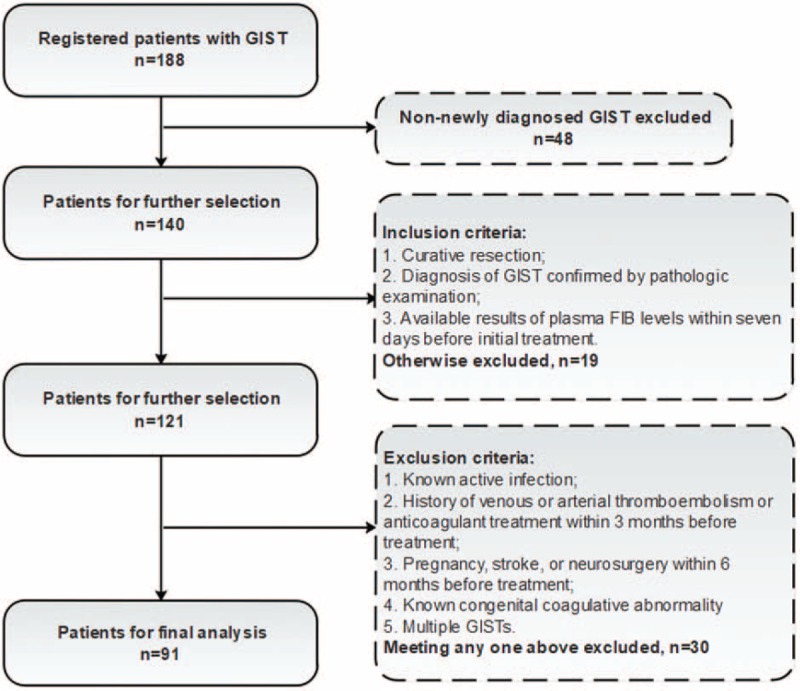
Patient selection flow.

We retrospectively reviewed patients with newly-diagnosed GISTs who underwent curative surgical resection at the First Affiliated Hospital of Xi’an Jiaotong University between January 2014 and March 2016. The inclusion criteria included: diagnosis of GIST confirmed by pathologic examination of resection specimen meeting Protocol for the Examination of Specimens From Patients With Gastrointestinal Stromal Tumor^[13]^; available results of plasma FIB within 7 days before initial treatment. The exclusion criteria were: known active infection when the baseline level of FIB was taken; a history of venous or arterial thromboembolism or anticoagulant treatment within 3 months before treatment; pregnancy, stroke, or neurosurgery within 6 months before treatment; known congenital coagulative abnormality; and multiple GISTs (Fig. 1 patient selection flow). Follow-up evaluations were performed every 3 months within the first year and every 6 months after the first year. Clinical check-up, radiological, and laboratory assessment was included in follow-up investigations.

Written informed consent for collecting medical information was obtained. Under Chinese law, this type of study, which does not involve any invasive investigation but relies on a retrospective analysis of patient files, does not need the approval of the institutional review board.

### 2.2 Statistical analysis

Count data were reported using absolute frequencies and percentages, and continuous variables were displayed by medians (25th–75th percentile). OS was defined as the time from the start of surgery to death. RFS was defined as a composite endpoint of recurrence or death, whatever came first. Kaplan–Meier methods was used to estimate OS, while competing risk estimators was employed to estimate the cumulative incidence of non-terminal endpoints (recurrence) accounting for mortality as the competing event. Log-rank tests and Gray tests were used to compare survivor functions and cumulative incidence functions, respectively. Cox regression was applied in multivariable modeling of time-to-endpoints.

We applied Cox regression to estimate the independent relationship between FIB and outcomes of mortality and recurrence after adjusting for potential confounders. We then explored the relationship between them using the smoothing plot. We further applied a 2-piecewise Cox regression model to examine the threshold effect of FIB on risk of mortality and recurrence according to the smoothing plot. An inflection of FIB, at which the relationship began to change and become eminent, was determined using a trial method. The latter was to move the trial inflection point along a pre-defined interval and detect the inflection point that gave the maximum model likelihood.

All statistical analysis was performed using R (http://www.R-project.org).

## 3 Results

Ninety-one patients were included in the study (Table 1). All patients underwent surgery of curative resection. No patients had evidence of distant metastasis at the time of surgery. cKIT immunohistochemistry was positive in all patients (100%). The median age at surgery was 60.0 years (range, 39–81). The median tumor size was 4.0 cm (range, 0.2–15). Thirty-two patients (35.2%) received adjuvant treatment of imatinib for 1 to 3 years according to the current guidelines. Fifteen (16.5%) patients had a second primary malignancy (SPM) before or during the study.

**Table 1:**
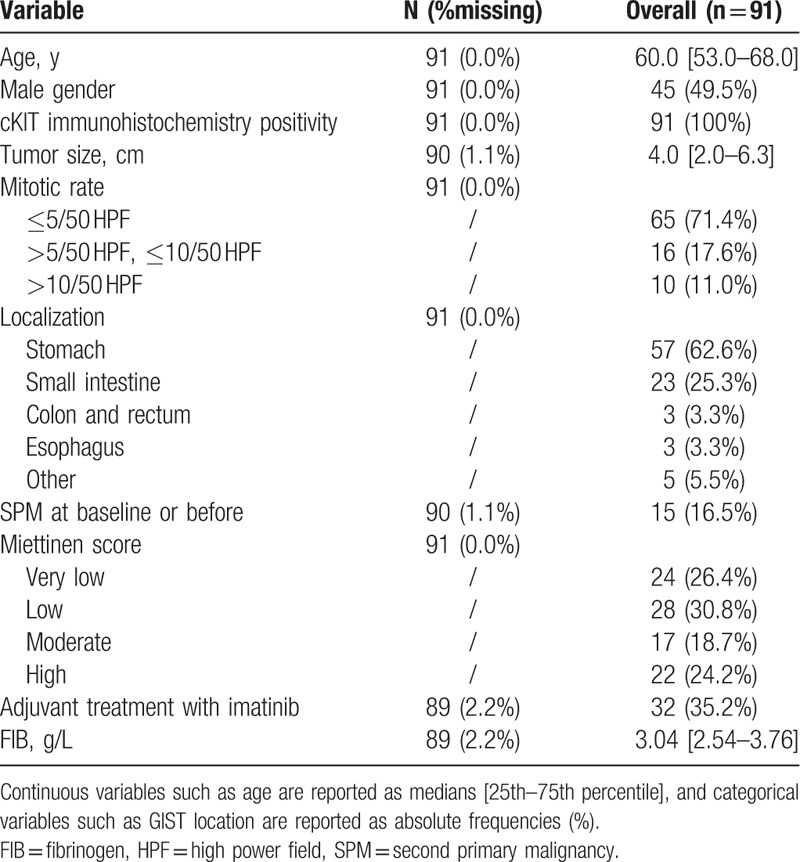
Baseline characteristics.

**Figure 2:**
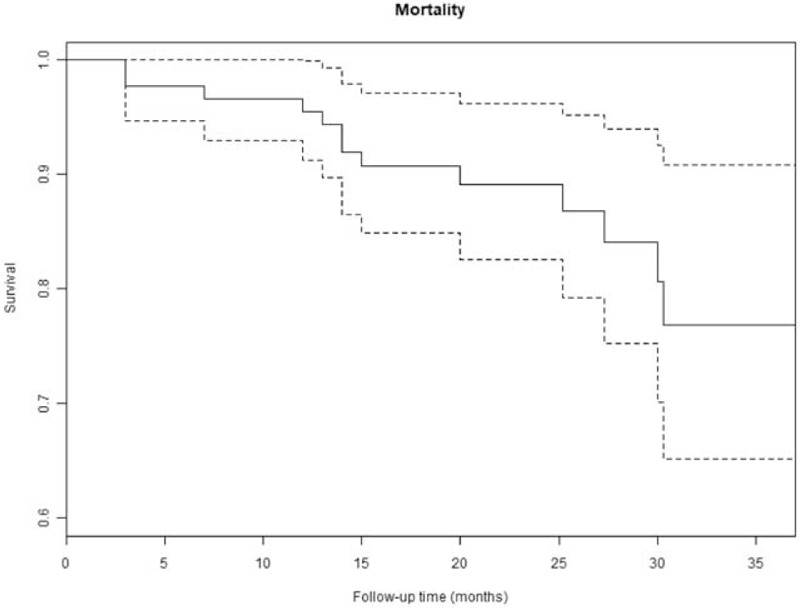
The Kaplan–Meier estimates of overall survival rates.

**Figure 3:**
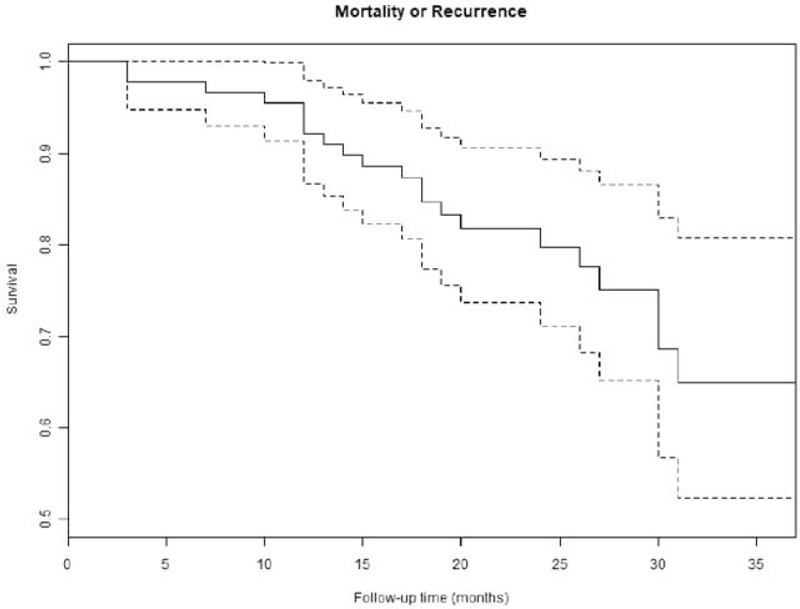
The Kaplan–Meier estimates of recurrence-free survival rates.

**Figure 4:**
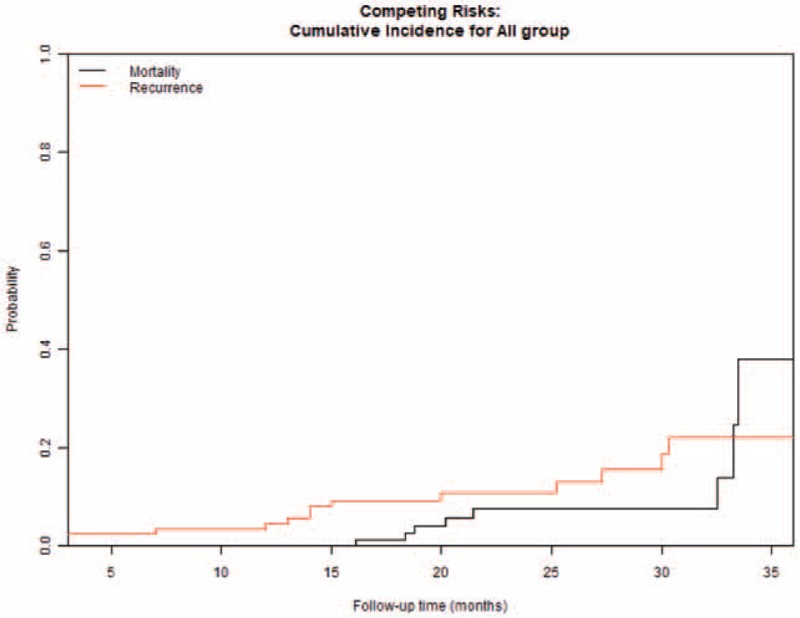
The cumulative recurrence rate accounting for death as a competing risk.

**Figure 5:**
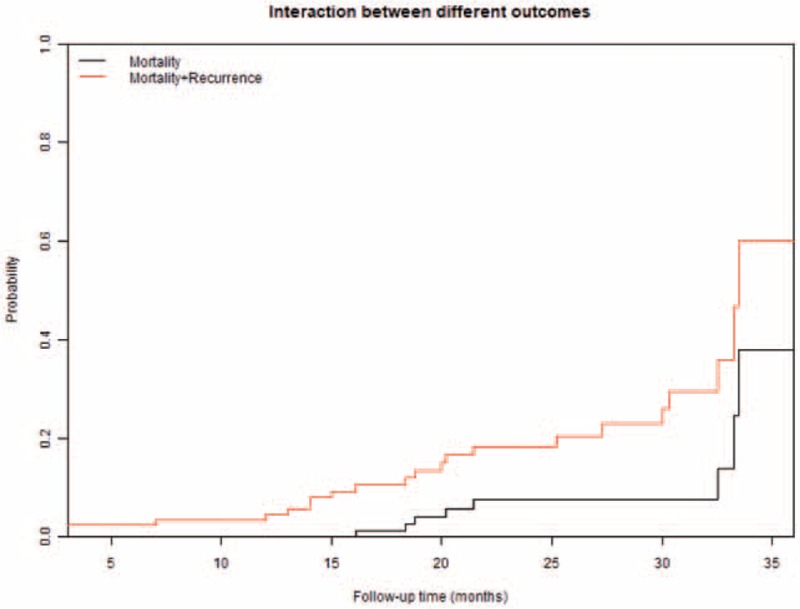
Interaction between recurrence and death risk using competing risk estimators.

Patients were followed from 13 months to 37 months (median: 2 years). Only 2 patients (2.2%) were lost-to-follow-up. Thirteen (14.2%) patients died during follow-up. The 1, 2, and 3 years overall survival rates using Kaplan–Meier method were 95.5% (95%CI: 91.3–99.9), 89.1% (95%CI: 82.5–96.2) and 76.9% (95%CI: 65.2–90.8), respectively (Fig. 2). The 1, 2, and 3 year recurrence-free survival rates using Kaplan–Meier method were 94.4% (95%CI: 89.7–99.3), 85.6% (95% CI: 77.8–94.1), and 75.6% (95% CI: 68.7–91.5), respectively (Fig. 3). Nine patients (9.8%) developed local recurrence in the follow-up. The cumulative 1, 2, and 3 years recurrence rates were estimated at 0% (95% CI: NaN), 7.4% (95% CI: 2.7–15.4), 24.5% (95% CI: 5.8–49.8) accounting for death as a competing risk (Figs. 4 and 5).

In multivariable Cox regression analysis (Table 2), we used 3 models to adjust for different confounders. Model 1 was unadjusted. Model 2 was adjusted for age at entry, sex, and SPM at baseline or before. And Model 3 was adjusted for variables in Model 2 plus adjuvant treatment with Imatinib and Miettinen risk score. We did not include tumor size, mitotic rate, and tumor location, because they are incorporated in the Miettinen score model. For the outcome of death, the results showed that 1 g/L increase in FIB level was associated with a 1.80-fold increase in the risk of death (HR = 1.80, 95% CI: 1.30–2.65, *P* = .001) in Model 1, 2.32-fold increase in the risk of death (HR = 2.32, 95% CI: 1.52–3.58, *P* < .001) in Model 2, and 2.02-fold increase in the risk of death (HR = 2.02, 95% CI: 1.33–3.21, *P* = .004) in Model 3. As for the outcome of death or recurrence, 1 g/L increase in FIB level was associated with a 1.73-fold increase in the risk of death or recurrence (HR = 1.73, 95% CI: 1.20–2.37, *P* = .001) in Model 1, 1.81-fold increase in the risk of death or recurrence (HR = 1.81, 95% CI: 1.37–2.50, *P* = .001) in Model 2, and 1.64-fold increase in the risk of death or recurrence (HR = 1.64, 95% CI: 1.11–2.43, *P* = .010) in Model 3.

**Table 2:**
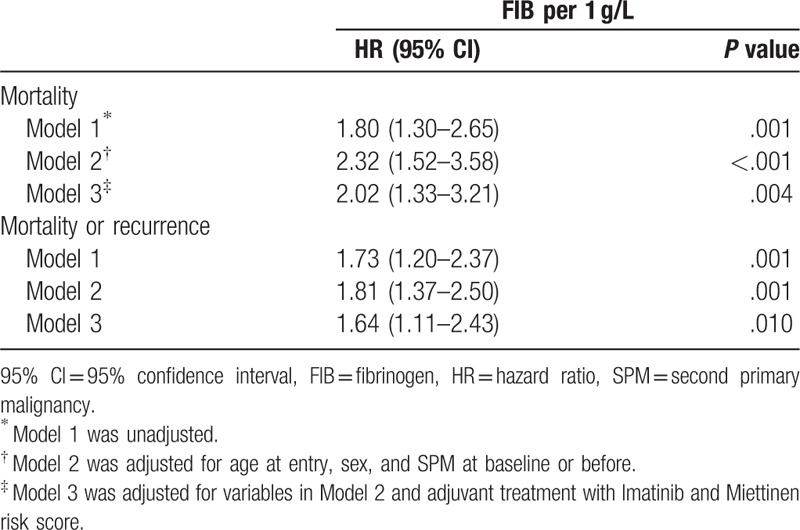
Associations of fibrinogen with risk of mortality and recurrence in the cohort.

**Figure 6:**
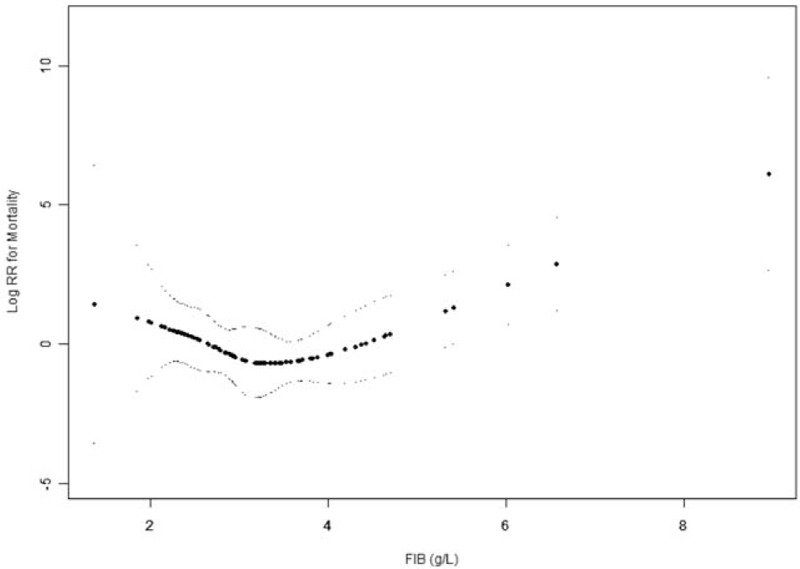
The relationship between FIB and the risk of death. A nonlinear relationship was observed after adjusting for confounding factors. A threshold for FIB of 3.7 g/L existed for the risk of death in GISTs patients. FIB = fibrinogen, GISTs = gastrointestinal stromal tumors.

**Figure 7:**
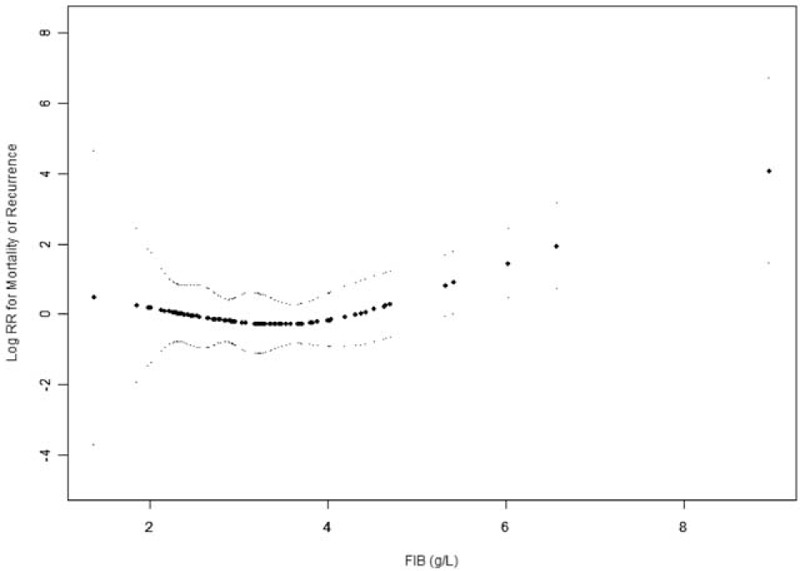
The relationship between FIB and the risk of death or recurrence. A nonlinear relationship was observed after adjusting for confounding factors. A threshold for FIB of 3.7 g/L existed for the risk of death or recurrence in GISTs patients. FIB = fibrinogen, GISTs = gastrointestinal stromal tumors.

We then explored the relationship between FIB and the outcomes using the smoothing plot. A nonlinear relationship was observed (Figs. 6 and 7). After applying a two-piecewise Cox regression model to examine the threshold effect of FIB on risk of outcomes, an inflection of FIB at 3.7 g/L was determined (Table 3). When FIB was above the inflection point (3.7 g/L), 1 g/L increase in FIB level was associated with a 3.90-fold increase in the risk of death (HR = 3.90, 95% CI = 1.90–8.15, *P* < .001), and the risk of death or recurrence also increased with the increase of FIB levels (HR = 2.43, 95% CI = 1.40–4.06, *P* = .002). When FIB level was <3.7 g/L, the association between FIB and risk of death (HR = 0.40, 95% CI = 0.10–1.44, *P* = .153) or between FIB and risk of death or recurrence (HR = 0.71, 95% CI = 0.22–1.83, *P* = .416) was not significant.

**Table 3:**
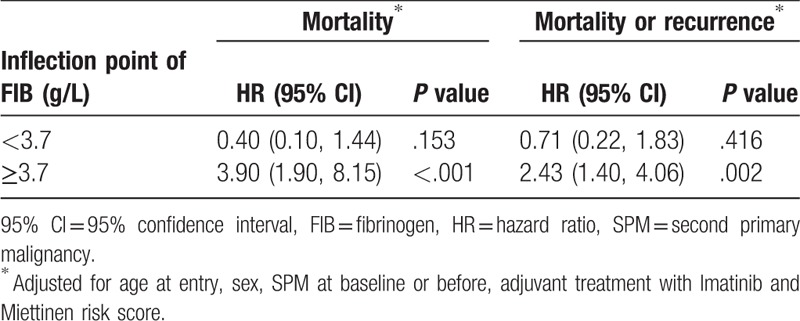
Threshold effect analysis of fibrinogen on mortality or recurrence using piece-wise Cox regression.

## 4 Discussion

In this study we examined the clinical course of 91 patients with GIST after curative surgery and found a significant association between high levels of FIB and decreased OS and RFS. And we discovered a nonlinear relationship between levels of FIB and the risk of death or recurrence. Further, we detected a threshold for FIB (3.7 g/L) on the prognosis of GISTs patients. When FIB was above the inflection point, the increase in FIB levels was strongly associated with increase in the risk of death or recurrence.

Evidence of coagulation activation is a common finding in many patients with tumors.^[14–17]^ Tumors contribute to a hypercoagulable state in many ways, including hemodynamic changes, acute phase response, tissue necrosis, and abnormal protein metabolism.^[17,18]^ However, the most important factors derive from the tumor cells themselves. Tumor cells can release procoagulant factors like tissue factor (TF), cancer procoagulant (CP), and tumor mucins and subsequently activate the hemostatic system. On the other hand, clotting cascade components and vascular factors associated with it play an essential role in tumor progression, angiogenesis, invasion, and metastasis. There are already animal and in vitro studies suggesting that an anti-neoplastic effect is exerted by anticoagulants, particularly the low molecular weight heparins (LMWHs), through several mechanisms, including interference with angiogenesis, tumor cell adhesion and invasion, formation of metastasis, and the immune system.

FIB (coagulation factor I) plays a vital role in the coagulation system. It bridges activated platelets and establishes a consolidating fibrin network by being the key substrate for thrombin. In previous studies, elevated levels of FIB have been reported to correlate with higher TNM staging and unfavorable prognosis in several types of malignancies. Poherauer et al^[8]^ reported that FIB plasma levels was an independent prognostic parameter in patients with cervical cancer. Takeuchi et al^[9]^ revealed that pretreatment plasma fibrinogen level correlated with tumor progression and metastasis in patients with carcinoma of the esophagus. Lee et al^[10]^ reported that plasma FIB level was an useful indicator in predicting the involvement of adjacent organ in patients with advanced gastric cancer. Son et al^[11]^ reported that preoperative plasma hyperfibrinogenemia was predictive of adverse prognosis in patients with nonmetastatic colon cancer. Despite the special property of the malignant potential of GISTs, our study results are in accordance with the previous studies, indicating elevated FIB as an independent prognostic marker for a worse clinical outcome in GIST patients.

At present, there is no ideal plasma tumor biomarker for GIST to evaluate the prognosis. FIB is a coagulation index which has been tested as a preoperative routine. It is easily-available with high sensitivity, small damage and low cost. Accordingly, FIB can assist in predicting the prognosis of GIST patients. Doctors should pay more attention to the patients with preoperative FIB level above 3.7 g/L and intervene in time when there is indication of progression.

There are some limitations in our study that need to be noted. Venous thromboembolism (VTE) events and some other potential confounding factors have not been assessed systematically in this study. And we cannot totally exclude a selection bias in this study due to the nature of retrospective design.

In conclusion, we found that high levels of FIB were independently associated with reduced OS and RFS. When FIB was ≥3.7 g/L, elevated FIB can serve as an independent prognostic biomarker for a worse clinical outcome in GIST patients.

## Author contributions

**Conceptualization:** Jing Lu.

**Data curation:** Jing Lu.

**Formal analysis:** Jing Lu.

**Funding acquisition:** Xiangming Che, Lin Fan.

**Investigation:** Jing Lu.

**Methodology:** Jing Lu.

**Project administration:** Jing Lu.

**Resources:** Lin Fan.

**Software:** Jing Lu.

**Supervision:** Xiangming Che, Lin Fan.

**Validation:** Shuangjiang Chen, Xuqi Li, Guanglin Qiu, Shicai He, Haijiang Wang, Libo Zhou, Yaheng Jing.

**Visualization:** Jing Lu.

**Writing** – **original draft:** Jing Lu.

**Writing** – **review and editing:** Jing Lu, Shuangjiang Chen, Xuqi Li, Guanglin Qiu, Shicai He, Haijiang Wang, Libo Zhou, Yaheng Jing, Lin Fan.
